# Environment-Induced Renormalization of Molecular Polarizabilities

**DOI:** 10.1021/acsphyschemau.6c00007

**Published:** 2026-05-13

**Authors:** Johannes Fiedler

**Affiliations:** Department of Physics and Technology, 1658 University of Bergen, Allégaten 55, 5007 Bergen, Norway

**Keywords:** macroscopic quantum electrodynamics, polarizability renormalization, Casimir−Polder interaction, cavity quantum electrodynamics, environment-induced spectroscopy, electromagnetic Green tensor, continuum solvation models

## Abstract

The electromagnetic environment modifies atomic properties through self-energy corrections arising from vacuum-field fluctuations shaped by material boundaries. While such effects are commonly discussed in terms of energy level shifts and decay rates, their influence on atomic response functions has received much less attention. Here, we investigate how environment-induced self-energy corrections renormalize the dynamic polarizability of an atom. Using macroscopic quantum electrodynamics, we analyze a hydrogen atom at the center of a spherical vacuum cavity embedded in a dielectric medium. The cavity-induced Lamb shifts of the atomic levels propagate into the frequency-dependent polarizability through the sum-over-states representation. We find that cavity confinement shifts absorptive resonances, modifies their amplitudes, and can generate additional geometry-induced spectral features within the finite range of cavity radii. The polarizability along the imaginary-frequency axis remains well-behaved, consistent with a passive electromagnetic environment. These results demonstrate that structured electromagnetic environments can qualitatively reshape atomic response functions and provide a route toward geometry-aware descriptions of molecular polarizabilities in complex media.

## Graphical abstract

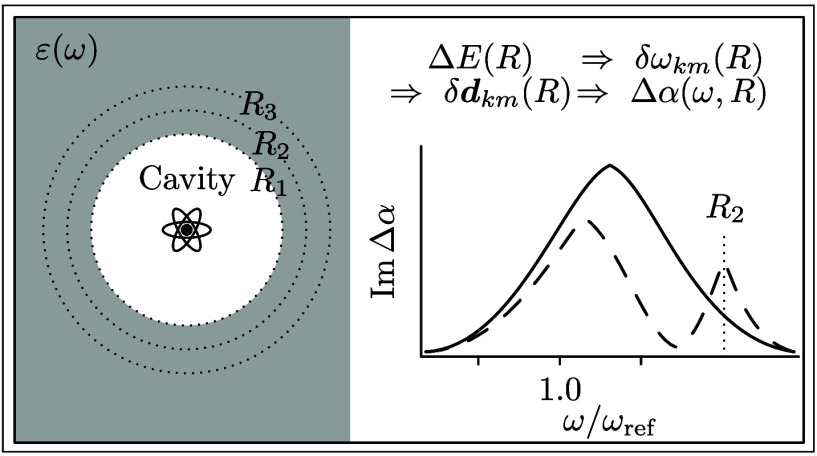

## 1 Introduction

The interaction between atoms and the electromagnetic vacuum is profoundly modified by the presence of macroscopic bodies. This modification underlies a wide range of phenomena, including the Lamb shift, changes in spontaneous-emission rates, and dispersion forces such as the Casimir–Polder interaction. Within macroscopic quantum electrodynamics (mQED), these effects are understood as self-energy corrections arising from the interaction of atomic charge and current distributions with vacuum field fluctuations shaped by material boundaries.^1–4^

Traditionally, environment-induced self-energy effects have been discussed primarily in terms of energy-level shifts and decay rates. Seminal theoretical works established how surfaces and cavities modify atomic transition frequencies and lifetimes through the electromagnetic Green tensor of the surrounding medium.^5–10^ In this picture, the Casimir–Polder interaction can be interpreted as a position-dependent Lamb shift, encoding the influence of geometry and material response on atomic energy levels.^2^ Vacuum-field-induced radiative coupling and state mixing have been discussed in closely related QED contexts, ranging from interference effects in multilevel atoms to spontaneous radiative coupling and environment-induced coherent dynamics.^11–14^ Here, we build on these ideas and focus on their consequences for frequency-dependent molecular response functions, in particular the dynamic polarizability. Closely related mixing of atomic and photonic degrees of freedom has also been extensively studied in cavity-QED systems operating in the ultrastrong-coupling regime, where hybrid light–matter states emerge from strong vacuum-field interactions.^15–17^ The relation between such cavity-QED effects and perturbative environment-induced modifications of atomic properties in macroscopic QED has been discussed in several recent reviews.^3^ In contrast, the present work focuses on the weak-coupling regime, where the electromagnetic environment modifies atomic properties through self-energy corrections, while atomic and photonic excitations remain well-defined.

In contrast, considerably less attention has been paid to how such self-energy corrections propagate into the *response properties* of atoms and molecules. In most practical descriptions, the electromagnetic environment is assumed to affect atomic energies while leaving intrinsic response functions, such as the dynamic polarizability, unchanged. This separation is implicit in a wide range of models used in molecular physics, quantum chemistry, and dispersion-force calculations, where fixed polarizabilities are combined with geometry-dependent field propagators.^18–20^

From a microscopic perspective, however, this separation is not entirely consistent. Any modification of atomic energy levels implies a corresponding modification of the underlying eigenstates. As a result, both the transition frequencies and the dipole matrix elements entering linear and nonlinear response functions are, in principle, renormalized by the electromagnetic environment. This observation raises the question of the extent to which environment-induced self-energy effects can qualitatively alter atomic and molecular response functions even in the absence of external driving fields.

Recent theoretical work has begun to explore environment-induced state mixing and its consequences for radiative processes, symmetry breaking, and effective transition moments.^21–23^ Related applications of the real-cavity model within macroscopic QED have recently been explored in the context of resonant energy transfer in structured environments, where the local electromagnetic environment can give rise to discriminatory transfer pathways.^24,25^ At the same time, polarizable continuum models (PCM) have been widely employed to describe how a surrounding medium modifies molecular energies by introducing an effective cavity that separates microscopic electronic degrees of freedom from macroscopic dielectric response.^26,27^ Despite their different origins, both approaches emphasize that geometry and material properties can feed back into the internal structure of quantum systems.

In this work, we combine these perspectives and investigate how cavity-induced self-energy corrections renormalize the atom’s dynamic polarizability. Rather than focusing on quantitative predictions for specific materials, our goal is to provide a transparent and controlled analysis of the underlying mechanisms. We therefore consider the hydrogen atom embedded at the center of a spherical vacuum cavity surrounded by a homogeneous dielectric medium, which serves as a minimal benchmark system.

Within macroscopic QED, we first derive the cavity-induced self-energy shift and show how it leads to state-dependent energy renormalization. We then analyze how these shifts propagate into the frequency-dependent polarizability through the sum-over-states representation. This approach allows us to identify two qualitatively distinct effects: a systematic shift of the dominant absorptive resonances and the appearance of additional geometry-induced response features absent in free space.

By focusing on hydrogen, all required matrix elements can be evaluated analytically, enabling a clear separation between universal geometric effects and the system-specific electronic structure. The resulting framework provides a conceptual bridge between cavity-modified vacuum fluctuations, polarizable continuum descriptions, and environment-engineered response functions. Beyond its role as a benchmark, the present analysis outlines a route toward incorporating self-energy-induced renormalization effects into effective descriptions of molecular response properties in complex electromagnetic environments.

## 2 Theoretical Framework

### 2.1 Casimir–Polder Interaction as Perturbative Self-Energy

The Casimir–Polder (CP) interaction originates from vacuum fluctuations of the electromagnetic field, which induce correlated dipole moments in polarizable particles. At the microscopic level, this interaction is governed by the electric–dipole coupling between the particle and the quantized electromagnetic field,

1 $${Ĥ}_{\mathrm{int}}=-\mathrm{d̂}\cdot Ê\left(r_{A}\right)$$

where ***d̂*** denotes the electric dipole operator of the atom or molecule and ***E* ^** is the electric field operator evaluated at the particle position ***r***_*A*_. Throughout this work, we employ the electric-dipole coupling in the multipolar (Power–Zienau–Woolley) gauge, which is the standard formulation used in macroscopic quantum electrodynamics.^28–30^

The total Hamiltonian of the coupled atom–field system is written as^2^

2 $$Ĥ={Ĥ}_{A}+{Ĥ}_{F}+{Ĥ}_{\mathrm{int}}$$

where *Ĥ*_*F*_ describes the electromagnetic field in the presence of absorbing macroscopic bodies and *Ĥ*_*A*_ is the Hamiltonian of the free atom, *Ĥ*_*A*_ = Σ_*n*_ℏω_*n*_|*n*⟩⟨*n*|. Since the CP interaction is weak, the Schrödinger equation can be solved perturbatively with the unperturbed Hamiltonian

3 $${Ĥ}_{0}={Ĥ}_{A}+{Ĥ}_{F}$$

which defines a product basis of atomic and field states, |φ⟩ = |φ_*A*_⟩ ⊗ |φ_*F*_⟩, reflecting the independence of atomic and field degrees of freedom in the absence of coupling. In the low-temperature limit, the unperturbed state is given by |φ_*m*_⟩ = |*m*, {0}⟩, meaning the particle is excited in state |*m*⟩, whereas the field remains in ground state.

The leading contribution to the interaction energy arises in second-order perturbation theory,

4 $$\Delta E_{m}=\underset{\varphi_{I}\neq \varphi_{m}}{\sum }\frac{\left\langle \varphi_{m}\right|{Ĥ}_{\mathrm{int}}\left|\varphi_{I}\right\rangle \left\langle \varphi_{I}\right|{Ĥ}_{\mathrm{int}}\left|\varphi_{m}\right\rangle }{E_{\varphi_{m}}-E_{\varphi_{I}}}$$

where the intermediate states |φ_*I*_⟩ = |*k*, **1**(***r***,ω)⟩ consist of an excited atomic state |*k*⟩ and a single elementary excitation of the electromagnetic field.

Evaluating the matrix elements using the macroscopic-QED expression for the electric field operator yields an energy shift of the form

5 ΔEm=−μ0π∑k∫0∞dωω2ωkm+ωdmk·Im⁡G(rA,rA,ω)·dkm

where **G** denotes the dyadic electromagnetic Green tensor and ***d***_*mk*_ = ⟨*m*| ***d̂*** |*k*⟩. For excited atomic states, the self-energy generally acquires both a real and an imaginary part, corresponding to energy shifts and spontaneous decay rates, respectively. In the following, we focus on the real part of the self-energy, which is obtained from the principal value of the frequency integral and gives rise to the Casimir–Polder level shift.

Equation 5 shows that the CP interaction is governed by the electromagnetic Green tensor evaluated in the coincidence limit with both spatial arguments taken at the particle position. Physically, this reflects the fact that the interaction arises from the emission and reabsorption of virtual photons by the same particle.

The Green tensor can be decomposed into a free-space contribution and a scattering part induced by the environment,

6 $$G\left(r,r^{\prime },\omega \right)=G^{\left(0\right)}\left(r,r^{\prime },\omega \right)+G^{\left(\mathrm{sc}\right)}\left(r,r^{\prime },\omega \right)$$

In the coincidence limit, the imaginary part of the free-space Green tensor is position-independent

7 Im⁡G(0)(r,r,ω)=ω6πcI

and is assumed to be included in the bare atomic spectrum. The resulting CP interaction arising from the free-space Green function yields the well-known vacuum Lamb shift. Consequently, the environment-dependent part of the CP interaction is entirely determined by the scattering Green tensor, **G** ^(sc)^. From this perspective, the Casimir–Polder interaction can be interpreted as a position-dependent Lamb shift arising from the modification of vacuum fluctuations by the electromagnetic environment. As in the usual derivation of the Lamb shift, the free-space contribution to the perturbative self-energy formally contains ultraviolet divergences. In practice, these divergences are absorbed into the renormalized atomic transition frequencies, and only the environment-dependent scattering part of the Green tensor contributes to observable energy shifts in the present treatment.

### 2.2 Environment-Induced Correction of Atomic States

Beyond the energy shift discussed above, the electromagnetic self-energy also induces corrections to the atomic eigenstates themselves. Within time-independent perturbation theory, the dressed atom–field state corresponding to an excited atomic level |*m*⟩ is given by

8 $$\left|{\mathrm{\varphi ̃}}_{m}\right\rangle =\left|\varphi_{m}\right\rangle +\left|\delta \varphi_{m}\right\rangle$$

where the first-order correction reads

9 $$\left|\delta \varphi_{m}\right\rangle =\underset{\varphi_{I}\neq \varphi_{m}}{\sum }\frac{\left\langle \varphi_{I}\right|{Ĥ}_{\mathrm{int}}\left|\varphi_{m}\right\rangle }{E_{\varphi_{m}}-E_{\varphi_{I}}}\left|\varphi_{I}\right\rangle$$

The intermediate states are of the form |φ_*I*_⟩ = |*k*, **1**(***r***,ω)⟩, corresponding to an atomic excitation |*k*⟩ accompanied by a single elementary excitation of the electromagnetic field.

Substituting the electric–dipole interaction *Ĥ*_int_ = −***d̂*** · ***Ê*** (***r***_*A*_) and using the macroscopic-QED representation of the electric field operator, the correction can be written as

10 $$\left|\delta \varphi_{m}\right\rangle =-\underset{k}{\sum }\int_{0}^{\infty }d\omega \frac{{\mathrm{d̂}}_{km}\cdot Ê\left(r_{A},\omega \right)}{\hbar \left(\omega_{km}+\omega \right)}\left|k,1\left(r,\omega \right)\right\rangle$$

This expression describes the virtual emission and reabsorption of the electromagnetic field via single-photon intermediate states and thus represents the state-level counterpart of the Casimir–Polder self-energy.

For the calculation of atomic response properties, it is convenient to eliminate the field degrees of freedom and work with an effective atomic Hamiltonian. Within second-order perturbation theory (equivalently, a Feshbach-projection or self-energy approach), the reduced atomic dynamics is governed by

11 $${Ĥ}_{\mathrm{eff}}\left(\omega \right)={Ĥ}_{A}+\mathrm{\sum ̂}\left(\omega \right)$$

with matrix elements Σ_*km*_(ω) given in eq 13 denoting the self-energy operator arising from virtual single-photon intermediate states.^4,31^ The (right) eigenstates of *Ĥ*_eff_ define the *effective dressed atomic states* that enter response functions. To the leading order in Σ, one obtains

12 $$\left|\mathrm{m̃}\right\rangle =\left|m\right\rangle +\underset{k\neq m}{\sum }\frac{\sum_{km}\left(\omega_{m}\right)}{E_{m}-E_{k}}\left|k\right\rangle$$

where the off-diagonal matrix elements of the self-energy operator are given by

13 ∑km(ωm)=−μ0π∫0∞dωω2ωkm+ωdkm·Im⁡G(rA,rA,ω)·dmk

For excited states, the self-energy is generally complex. In the following, we restrict ourselves to the real part of Σ_*km*_, which describes coherent state mixing associated with Lamb-shift-type corrections. The imaginary part, related to spontaneous decay processes, is not considered here.

Equation 12 shows explicitly that the electromagnetic environment induces a coherent mixing of atomic eigenstates rather than merely shifting their energies. This environment-induced state mixing forms the basis for the renormalization of molecular response functions discussed in the following sections.

### 2.3 Environment-Induced Dipole Expectation Values and Selection Rules

The environment-induced state mixing derived above has direct consequences for the expectation value of the electric dipole operator. While the dipole moment of an isolated nonpolar atom or molecule vanishes in any stationary eigenstate,

14 ⟨m|d̂|m⟩=0

this property no longer holds once the electromagnetic environment induces a coherent admixture of other atomic states. The expectation value of the dipole operator is evaluated in the effective atomic state obtained after eliminating the electromagnetic field degrees of freedom. Consequently, the induced dipole moment originates from environment-induced mixing of atomic eigenstates rather than from matrix elements between different photon-number sectors.^2,4^

Using the dressed atomic state |*m̃*⟩ = |*m*⟩ + Σ_*k*≠*m*_ *c*_*km*_|*k*⟩ with coefficients *c*_*km*_ = Σ_*km*_(ω_*m*_)/(*E*_*m*_ – *E*_*k*_), the expectation value of the dipole operator becomes

15 $$\left\langle \mathrm{d̂}\rangle_{m}=\left\langle \mathrm{m̃}\right|\mathrm{d̂}\right|\mathrm{m̃}\rangle =\underset{k\neq m}{\sum }\left(c_{km}d_{mk}+c_{km}^{*}d_{km}\right)+O\left(c^{2}\right)$$

To the leading order in the self-energy, the environment induces a static dipole moment even for originally nonpolar states.

Equation 15 shows that the induced dipole moment is a purely quantum effect arising from coherent virtual transitions between atomic eigenstates mediated by the electromagnetic environment.

The appearance of a nonzero dipole expectation value is governed by strict selection rules. Since the electric dipole operator is a spherical tensor of rank one, individual dipole matrix elements ***d***_*mk*_ obey the usual selection rules

16 Δl=±1,⁣Δm=0,±1

together with opposite parity of the participating states.

However, the environment-induced dipole expectation value involves products of dipole operators mediated by the electromagnetic Green tensor. As a consequence, the resulting operator structure transforms as a tensor of rank up to two. This allows for induced dipole moments with

17 Δm=0,±2

Physically, this reflects the tensor structure generated by virtual two-dipole processes mediated by the electromagnetic field rather than a genuine electric quadrupole interaction.

A useful consistency check is obtained by switching off the environment. In the absence of macroscopic bodies, the scattering Green tensor vanishes, **G** ^(sc)^ → 0, such that the off-diagonal self-energy matrix elements Σ_*km*_ (and hence the mixing coefficients *c*_*km*_ = Σ_*km*_/(*E*_*m*_ – *E*_*k*_)) disappear. Consequently, the dressed state reduces to the bare eigenstate, |*m̃*⟩ → |*m*⟩, and the induced dipole moment vanishes. In this limit, only the standard electric-dipole selection rules apply.

### 2.4 Environment-Induced Renormalization of the Polarizability

The environment-induced state mixing discussed above has direct consequences for the linear optical response. In free space, the dynamic electric polarizability associated with an atomic eigenstate |*m*⟩ is given by

18 $$\alpha_{ij}^{\left(m\right)}\left(\omega \right)=\frac{1}{\hbar }\underset{k}{\sum }\left(\frac{d_{i}^{mk}d_{j}^{km}}{\omega_{km}-\omega }+\frac{d_{j}^{mk}d_{i}^{km}}{\omega_{km}+\omega }\right)$$

which assumes stationary atomic eigenstates.

When the environment-induced state correction |*m*⟩ → |*m̃*⟩ is taken into account, the polarizability acquires an additional contribution,

19 $$\alpha_{ij}^{\left(m\right)}\left(\omega \right)\to \alpha_{ij}^{\left(m\right)}\left(\omega \right)+\Delta \alpha_{ij}^{\left(m\right)}\left(\omega \right)$$

where the correction Δα arises from both modified dipole matrix elements and shifted transition frequencies.

To the leading order in the self-energy, the wave function contribution to the polarizability correction is obtained by inserting |*m̃*⟩ = |*m*⟩ + Σ_*c*≠*m*_ *c*_*cm*_|*c*⟩ into eq 18 and retaining only linear terms in the mixing coefficients. This yields

20 $$\Delta \alpha_{\mathrm{ij},\mathrm{wf}}^{\left(m\right)}\left(\omega \right)=\frac{1}{\hbar }\underset{k,c\neq m}{\sum }\left[\frac{c_{cm}d_{i}^{ck}d_{j}^{km}+c_{cm}^{*}d_{i}^{mk}d_{j}^{kc}}{\omega_{km}-\omega }+\frac{c_{cm}d_{j}^{ck}d_{i}^{km}+c_{cm}^{*}d_{j}^{mk}d_{i}^{kc}}{\omega_{km}+\omega }\right]$$

At this stage, the correction involves a double sum over the intermediate state index *k* and the admixed state index *c*. Upon inserting the explicit expression for the mixing coefficients *c*_*cm*_ = Σ_*cm*_/(*E*_*m*_ – *E*_*c*_), the self-energy itself introduces additional sums and frequency integrals, yielding the expected higher-fold summation structure. This reflects the fact that the renormalized polarizability is a higher-order response quantity constructed from the same virtual processes underlying the Casimir–Polder interaction.

In addition to the wave function contribution, the polarizability also acquires a correction due to the shift of the transition frequencies, ω_*km*_→ ω_*km*_ + Δω_*km*_ with Δω_*km*_ = (Δ*E*_*k*_ – Δ*E*_*m*_)/ℏ. To the first order, this yields the energy-denominator contribution

21 $$\Delta \alpha_{\mathrm{ij},\mathrm{en}}^{\left(m\right)}\left(\omega \right)=-\frac{1}{\hbar }\underset{k}{\sum }\left[\frac{\Delta \omega_{km}}{{\left(\omega_{km}-\omega \right)}^{2}}d_{i}^{mk}d_{j}^{km}+\frac{\Delta \omega_{km}}{{\left(\omega_{km}+\omega \right)}^{2}}d_{j}^{mk}d_{i}^{km}\right]$$

The total correction to the polarizability is thus given by Δα = Δα_wf_ + Δα_en_. In the absence of the environment, **G** ^(sc)^ → 0 implies Σ_*cm*_ → 0 and Δω_*km*_ → 0, such that both contributions vanish and the standard free-space eq[Disp-formula eq18] is recovered.

## 3 Methods

All calculations are performed within macroscopic quantum electrodynamics in the electric-dipole approximation using the multipolar (Power–Zienau–Woolley) gauge. The electromagnetic environment is described via the scattering part of the dyadic Green tensor.

Hydrogenic wave functions are used in analytic form, and transition dipole matrix elements are obtained from radial integrals evaluated by numerical quadrature. The dynamic polarizability is computed using a sum-over-states approach with precomputed transition energies and dipole matrix elements.

The dielectric response of the surrounding medium is modeled by a Lorentz-type permittivity. All numerical calculations were performed using custom Python routines.

## 4 Numerical Examples

The purpose of this section is to provide a comprehensive understanding of the effects of different types of additives on the performance of PEMs. First, we provide minimal model systems that illustrate when environment-induced state mixing is absent and when it becomes operative. Second, we quantify the resulting renormalization of response functions for simple dispersive environments, using the simplest possible material model.

Throughout this section, we consider an isotropic, nonmagnetic medium described by an undamped Lorentz oscillator model for the permittivity,

22 $$\varepsilon \left(\omega \right)=1+\frac{{\omega_{p}}^{2}}{{\omega_{0}}^{2}-\omega^{2}}$$

and we focus on the scattering part of the Green tensor. For transparency, we work in the near-field (nonretarded) regime, where the scattering Green tensor in front of a planar dielectric half-space admits a simple closed form, and the physical role of the reflection amplitude becomes explicit. The fully retarded formulation can be incorporated without conceptual changes and is not required for the main message of this section.

### 4.1 A Two-Level System in Front of a Dielectric Plate

For the numerical example discussed above, we consider a strict two-level system with an optical transition frequency ω_*eg*_ = 2π × 5 × 10^14^ rad s^–1^ (corresponding to a wavelength of approximately 600 nm) and a dipole matrix element of magnitude *d*_*ge*_ = 3 × 10^–29^ C m, representative of a typical allowed electric-dipole transition in atoms or small molecules. The resulting static polarizability of the ground state, α(0) = 2*d*_*ge*_^2^/(ℏω_*eg*_), is of the order 10^–38^ C^2^ m^2^ *J* ^–1^, consistent with atomic-scale polarizabilities.

We consider a particle at a distance *z* in front of a planar dielectric half-space. In the nonretarded limit, the scattering Green tensor at coincident points is diagonal and can be expressed in terms of the electrostatic reflection amplitude

23 $$r\left(\omega \right)=\frac{\varepsilon \left(\omega \right)-1}{\varepsilon \left(\omega \right)+1}$$

This yields a compact expression for the Green function^32^

24 $$G^{\left(\mathrm{sc}\right)}\left(z,\omega \right)=\frac{c^{2}}{32\pi \omega^{2}z^{3}}r\left(\omega \right)\;\mathrm{diag}\left(1,1,2\right)$$

and therefore for the environment-induced level shifts and the resulting denominator correction Δα_en_. The dielectric half-space is modeled by an undamped Lorentz oscillator with resonance frequency ω_0_ = 2π × 3 × 10^15^ rad s^–1^ and oscillator strength ω_*p*_ = 2π × 2 × 10^15^ rad s^–1^. In the static limit, this corresponds to a relative permittivity ε(0) = 1 + ω_*p*_^2^/ω_0_^2^ ≈ 1.44, representative of a weakly polarizable dielectric material.

Figure 1 shows the environment-induced correction to the ground-state polarizability arising from the renormalization of the transition frequency, ω_*eg*_ → ω_*eg*_ + Δω_*eg*_(*z*), for several distances *z* from the surface. For a strict two-level system, the off-diagonal self-energy vanishes, such that the wave function contribution Δα_wf_ is identically zero and the leading correction is given solely by the denominator term Δα_en_.

**Figure 1:**
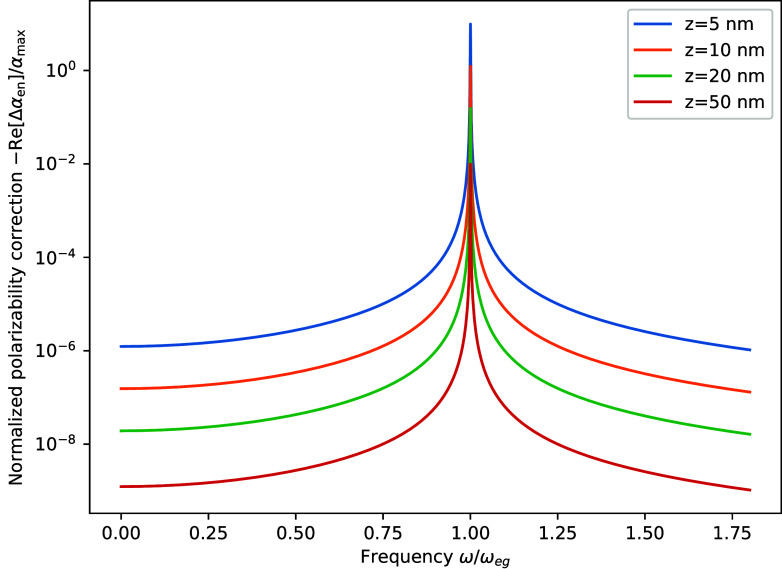
Environment-induced correction to the ground-state polarizability of a two-level system in front of a dielectric plate. The plotted quantity is the real part of the denominator contribution −*Re*Δα_en_(ω), normalized to the maximum of the bare resonant polarizability. Results are shown for distances *z* = 5, 10, 20, and 50 nm. The logarithmic scale highlights the small magnitude of the effect and its enhancement near the optical resonance.

To visualize this effect, the real part of the correction Δα_en_(ω) is normalized to the maximum of the imaginary part of the bare resonant polarizability, α_max_ = max_ω_|*Im* α_bare_(ω)|. This provides a natural, dimensionless reference scale set by the strength of the optical resonance.

As shown in Figure 1, the correction exhibits a strong frequency dependence and becomes comparable in magnitude to the peak polarizability in the immediate vicinity of the optical resonance for nanometer-scale distances. This behavior reflects the resonant enhancement of the denominator contribution Δα_en_, which is particularly sensitive to environment-induced shifts of the transition frequency. Away from resonance, the correction rapidly decreases and remains small compared to the bare response.

Importantly, this pronounced resonant modification occurs even though the wave function contribution Δα_wf_ vanishes identically for a strict two-level system. The result, therefore, demonstrates that substantial environment-induced renormalization of optical response functions can arise from transition-frequency shifts alone without invoking coherent-state mixing.

### 4.2 A Three-Level System in Front of a Dielectric Plate

The strict two-level model of Section 4.1 serves as a useful baseline: it demonstrates that sizable modifications of the resonant response can already arise from transition-frequency renormalization, even when coherent state mixing is absent. However, a closed two-level manifold is generally insufficient to capture the full mechanism of environment-induced state mixing discussed in Sections 2.2–2.4 because the off-diagonal self-energy responsible for coherent mixing may vanish identically within the restricted Hilbert space.

The minimal extension that generically enables environment-induced mixing is a three-level system. Here, we consider a Λ-type configuration consisting of two long-lived states |*g*⟩ and |*e*⟩ coupled via an auxiliary state |*a*⟩. We assume that the direct dipole matrix element between |*g*⟩ and |*e*⟩ vanishes by symmetry, ***d***_*ge*_ = 0, while ***d***_*ga*_ ≠ 0 and ***d***_*ea*_ ≠ 0 are allowed. This setting captures the essential physics while keeping the algebra fully transparent.

In this model, the environment generates an effective coherent coupling between |*g*⟩ and |*e*⟩ through virtual two-dipole processes involving |*a*⟩. Indeed, the off-diagonal self-energy acquires a nonzero contribution of the form

25 ∑ge(ωe)∝dga·Im⁡G(rA,rA,ω)·dae+...

where the omitted terms arise from the full frequency integration and the corresponding energy denominators. Consequently, the dressed eigenstates develop admixtures of opposite parity,

26 $$\left|ẽ\right\rangle =\left|e\right\rangle +\frac{\sum_{ge}\left(\omega_{e}\right)}{E_{e}-E_{g}}\left|g\right\rangle +...,,\left|\mathrm{g̃}\right\rangle =\left|g\right\rangle +\frac{\sum_{eg}\left(\omega_{g}\right)}{E_{g}-E_{e}}\left|e\right\rangle +...$$

which induces a finite static dipole expectation value and, importantly, a nonzero wave function contribution Δα_wf_ to the polarizability renormalization.

In the following, we evaluate these effects for the same planar Lorentz medium as in Section 4.1 using the nonretarded scattering Green tensor in eq 24. We focus on (i) the distance scaling of the induced coherent coupling and (ii) the relative magnitude of Δα_wf_ compared to the purely denominator-induced correction Δα_en_.

Figure 2 shows the wave function contribution Δα_wf_(ω) to the polarizability renormalization of a minimal three-level Λ system, evaluated for several atom–surface distances. In contrast to the strict two-level model, the auxiliary level enables a coherent admixture of opposite-parity states, which generates a nonvanishing Δα_wf_. While this contribution remains small compared to the bare resonant response at mesoscopic separations, it provides a clean proof of principle that environment-induced state mixing produces additional, symmetry-allowed response channels that are absent in a closed two-level manifold.

**Figure 2:**
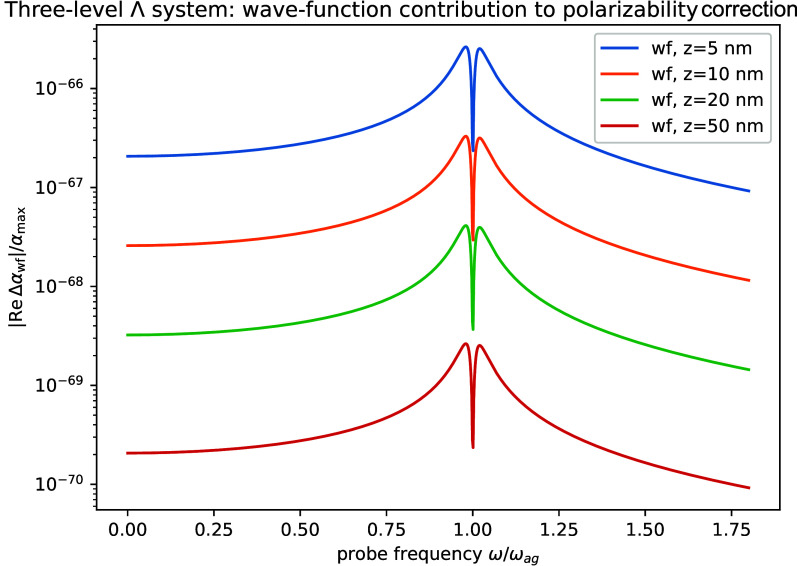
Wave function contribution Δα_wf_(ω) to the polarizability correction of a three-level Λ system in front of a dielectric plate, shown for several distances *z*. The signal is concentrated around the optical resonance and decays rapidly with increasing distance.

A direct physical consequence of the same mixing mechanism is the appearance of a finite transition dipole moment between states that are dipole-forbidden in free space. Figure 3 plots the induced matrix element |*d̃*_*ge*_|, which becomes nonzero due to the environment-induced admixture of the auxiliary state. Importantly, |*d̃*_*ge*_| increases rapidly as the separation approaches the molecular binding range. In this regime, the induced transition dipole can become comparable to typical allowed dipole moments, implying that nominally forbidden radiative channels may acquire appreciable oscillator strength. This effect is particularly relevant for surface-assisted decay and relaxation processes in the near field, where modified selection rules and additional decay pathways can impact spectroscopy and quenching dynamics.

**Figure 3:**
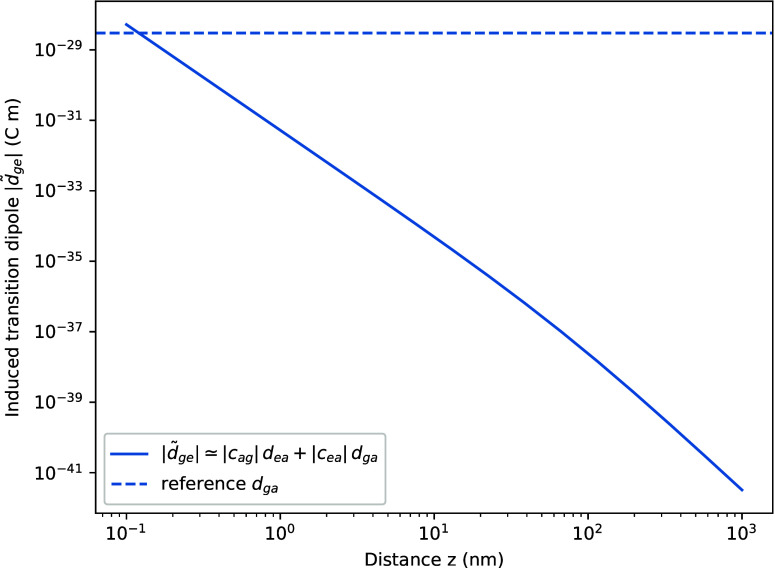
Induced transition dipole moment |*d̃*_*ge*_|for a transition that is dipole-forbidden in free space (*d*_*ge*_ = 0 in the bare basis). The environment-induced admixture of the auxiliary level generates a finite |*d̃*_*ge*_|, which grows strongly upon approaching the surface.

These strong short-range enhancements motivate the spherical-cavity geometry considered next, where the electromagnetic response surrounds the emitter and the relevant atom–medium separations can be systematically pushed into the subnanometer regime.

In realistic atomic or molecular systems, the polarizability arises from many dipole-allowed transitions rather than from a single dominant resonance. In such multilevel systems, environment-induced self-energy corrections modify the entire transition spectrum and therefore affect the response function through the combined shifts and mixing of many intermediate states. In particular, near-degenerate levels can lead to enhanced state mixing and, correspondingly, stronger modifications of the spectral response. As a result, the spectral features discussed above for simple model systems generally appear as superpositions of shifted and renormalized resonances. To illustrate this situation explicitly, we now consider the hydrogen atom as a minimal multilevel benchmark system.

### 4.3 Hydrogen Atom in a Spherical Cavity

Finally, we return to the hydrogen atom in a spherical real cavity geometry. This case provides a fully analytical benchmark for the scattering Green tensor at the cavity center and allows us to connect the general renormalization mechanism developed above to a paradigmatic quantum system with well-controlled spectroscopy. We use this example to validate limiting cases and to illustrate how the renormalized polarizability connects to geometry-induced self-energy corrections in a fully dispersive setting.

#### 4.3.1 Scattering Green Tensor of the Spherical Cavity

We consider an atom placed at the center of a spherical vacuum cavity of radius *R*, embedded in a homogeneous, isotropic dielectric medium characterized by the frequency-dependent permittivity ε­(ω). This geometry corresponds to the standard cavity construction employed in polarizable continuum models and macroscopic quantum electrodynamics.

For a source and observation point located at the cavity center, the scattering part of the electromagnetic Green tensor is isotropic and admits a closed-form expression:^8,33,34^

27 $$G_{\mathrm{sc}}\left(0,0,\omega \right)=\frac{i\omega }{6\pi c}C\left(\omega \right)I$$

The coefficient *C*(ω) encodes the electromagnetic reflection properties of the spherical vacuum–dielectric interface and depends solely on the cavity radius *R* and the material response ε­(ω). For small cavity radii, it can be expanded as^35,36^

28 $$C\left(\omega \right)=3\frac{\varepsilon \left(\omega \right)-1}{2\varepsilon \left(\omega \right)+1}\frac{c^{3}}{i\omega^{3}R^{3}}+\frac{9}{5}\frac{4\varepsilon^{2}\left(\omega \right)-3\varepsilon \left(\omega \right)-1}{{\left[2\varepsilon \left(\omega \right)+1\right]}^{2}}\frac{c}{i\omega R}+9\frac{\varepsilon^{5/2}\left(\omega \right)}{{\left[2\varepsilon \left(\omega \right)+1\right]}^{2}}-1+O\left(\frac{\omega R}{c}\right)$$

Equation 27 corresponds to the coincident-point limit of the Green tensor inside the cavity and already incorporates the full multipole structure of the electromagnetic boundary-value problem. Due to the vectorial nature of the electromagnetic field, only the electric dipole (l=1) contribution survives at the cavity center, while all higher multipole channels vanish identically by symmetry.

#### 4.3.2 Analytical Scaling Laws

Substituting eq 27 into the general macroscopic-QED expression for the self-energy shift, eq 13, yields

29 $$\Delta E_{a}=\frac{\hbar \mu_{0}}{6\pi^{2}c}\int_{0}^{\infty }d\xi \xi^{3}\alpha_{a}\left(i\xi \right)C\left(i\xi \right)$$

In the nonretarded regime relevant for cavity radii that are large compared to the atomic size, but small compared to the characteristic wavelengths, *C*(*i*ξ) reduces to the dipolar reflection amplitude of a spherical vacuum–dielectric interface,

30 $$C\left(i\xi \right)\approx \frac{3c^{3}}{\xi^{3}R^{3}}\frac{\varepsilon \left(i\xi \right)-1}{2\varepsilon \left(i\xi \right)+1}$$

The leading cavity-induced level shift, therefore, scales as

31 $$\Delta E_{a}\propto -\frac{1}{R^{3}}$$

corresponding to a cavity-induced Lamb shift arising from the modification of vacuum fluctuations by the dielectric boundary.

Finite-size effects of the atomic charge distribution and higher-order multipole corrections can be incorporated systematically but do not contribute to the leading order for an atom located at the cavity center.^35,36^

#### 4.3.3 Hydrogen Atom in a Spherical Cavity

To make the cavity-induced effects explicit, we now focus to the hydrogen atom. The unperturbed electronic eigenstates are given by the well-known hydrogenic wave functions,

32 $$\psi_{nlm}\left(r\right)=R_{nl}\left(r\right)Y_{lm}\left(\theta ,\phi \right)$$

with analytic radial functions $R_{nl}\left(r\right)$ and spherical harmonics $Y_{lm}$ . All required matrix elements can therefore be evaluated in closed form.

The electric-dipole operator couples states with Δl=±1 and Δ*m* = 0, ±1. The corresponding transition dipole moments read

33 $$d_{nlm,n\prime l\prime m\prime }=-e\left\langle nlm\right|r\left|n^{\prime }l^{\prime }m^{\prime }\right\rangle$$

where the radial integrals are known analytically and scale with the Bohr radius *a*_0_. Explicit expressions can be found, e.g., in refs 37 and 38 . Throughout this work, electric-dipole transition matrix elements are expressed in terms of reduced matrix elements in the sense of the Wigner–Eckart theorem. Specifically, we employ $\left\langle nl∥\mathrm{d̂}∥n^{\prime }l^{\prime }\right\rangle$ , which factorizes the angular-momentum dependence from the radial integrals and is independent of the magnetic quantum numbers. This representation is natural for isotropic response functions, such as the *m*-averaged dynamic polarizability considered here. For reference, the dipole matrix element associated with a specific spherical component *q* = 0, ±1 is obtained from the reduced matrix element by the standard angular factor $1/\sqrt{2l+1}$ .

All hydrogenic dipole matrix elements entering the numerical examples are computed using analytic hydrogenic radial wave functions and a numerical quadrature for the remaining radial integrals. For dominant transitions, this procedure reproduces known closed-form results to the percent level (e.g., the reduced 1s ↔ 2p matrix element agrees within ∼1%), while a few-percent deviations can occur for weak high-lying transitions due to the finite radial cutoff and quadrature tolerances. Since the present work aims at a qualitative assessment of cavity-induced trends rather than high-precision spectroscopy, this accuracy is sufficient.

The dynamic polarizability of a hydrogenic eigenstate follows directly from the sum-over-states representation,

34 $$\alpha_{nl}\left(\omega \right)=\frac{2}{\hbar }\underset{n^{\prime }l^{\prime }}{\sum }\frac{\omega_{n\prime l\prime ,nl}|d_{nl,n\prime l\prime }{|}^{2}}{{\omega_{n\prime l\prime ,nl}}^{2}-\omega^{2}}$$

which converges rapidly and can be evaluated numerically with high accuracy.

Within a polarizable continuum model, the cavity radius *R* is interpreted as the effective boundary separating the microscopic electronic degrees of freedom from the macroscopic dielectric response of the environment. For hydrogen, a physically motivated choice is *R* ∼ (1–3)*a*_0_, corresponding to the spatial extent of the electronic wave function. This choice avoids any reference to density-functional calculations and is sufficient to capture the leading cavity-induced self-energy corrections. We stress that the cavity radius *R* is introduced here as an electromagnetic (real-cavity) boundary in the sense of PCM-type models, not as a hard-wall confinement of the electronic wave function; no boundary condition is imposed on $\psi_{nlm}\left(r\right)$ at *r* = *R*.^8^

##### 4.3.3.1 Cavity-Induced Level Shifts

We first quantify the magnitude and scaling of the cavity-induced Lamb shift for several low-lying hydrogenic levels. Figure 4 shows the energy shifts Δν = Δ*E*/*h* for the states 1s, 2s, 2p, 3s, 3p, and 3d as a function of the cavity radius *R* in units of the Bohr radius. All shifts exhibit the characteristic nonretarded scaling Δ*E* ∝ *R* ^–3^ over the entire range shown, as expected from eq 29 together with the leading term of *C*(ω) in the small-cavity expansion. In the physically relevant PCM window *R* ≃ (1–3)*a*_0_ (gray band), the predicted shifts reach the range Δν ∼ 10^14^–10^17^ Hz, implying that cavity confinement can renormalize optical transition frequencies by amounts comparable to typical atomic line width and detuning scales. The ordering between different orbital states reflects the dependence on their dipole-coupled manifolds and associated dipole-strength sums entering the self-energy.

**Figure 4:**
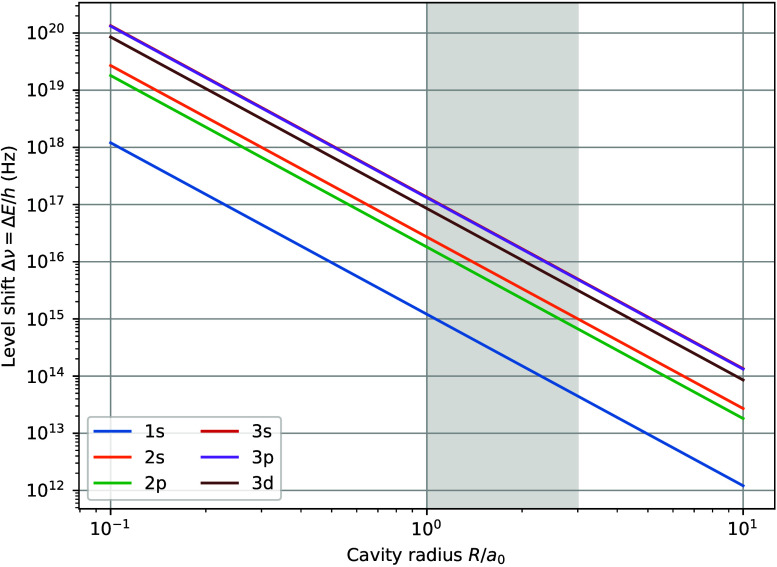
Cavity-induced level shifts of selected hydrogenic states (1s, 2s, 2p, 3s, 3p, 3d) as a function of the spherical cavity radius *R*. The shaded region highlights the PCM-relevant range *R* ≃ (1–3)*a*_0_.

##### 4.3.3.2 Renormalization of the Absorptive Response and Tracked Resonances

The level shifts shown above translate into a renormalization of the frequency-dependent polarizability through the perturbed transition frequencies in the sum-over-states expression. To visualize this effect, we consider the correction Δα­(ω) obtained by replacing ω_*km*_ → ω_*km*_ + δω_*km*_(*R*) in the denominators, where δω_*km*_(*R*) = [Δ*E*_*k*_(*R*) – Δ*E*_*m*_(*R*) ]/ℏ, while keeping the dipole matrix elements fixed (denominator correction). Figure 5 shows *Im* Δα­(ω) for the ground state 1s for several cavity radii. The dominant absorptive feature close to ω ≃ ω_ref_ corresponds to the main dipole-allowed resonance of the 1s state (dominated by the 1s → 2p channel). Importantly, the peak position shifts systematically with *R*, providing a direct spectroscopic signature of the cavity-induced Lamb shift. This behavior is quantified in the lower-left inset, where the tracked peak position ω_peak_(*R*) is shown together with the corresponding peak amplitude |*Im* Δα­(ω_peak_)|. Both quantities change appreciably across the PCM-relevant range *R* ≃ (1–3)*a*_0_, demonstrating that the cavity does not merely shift transition frequencies but can also reshape the absorptive response near resonance.

**Figure 5:**
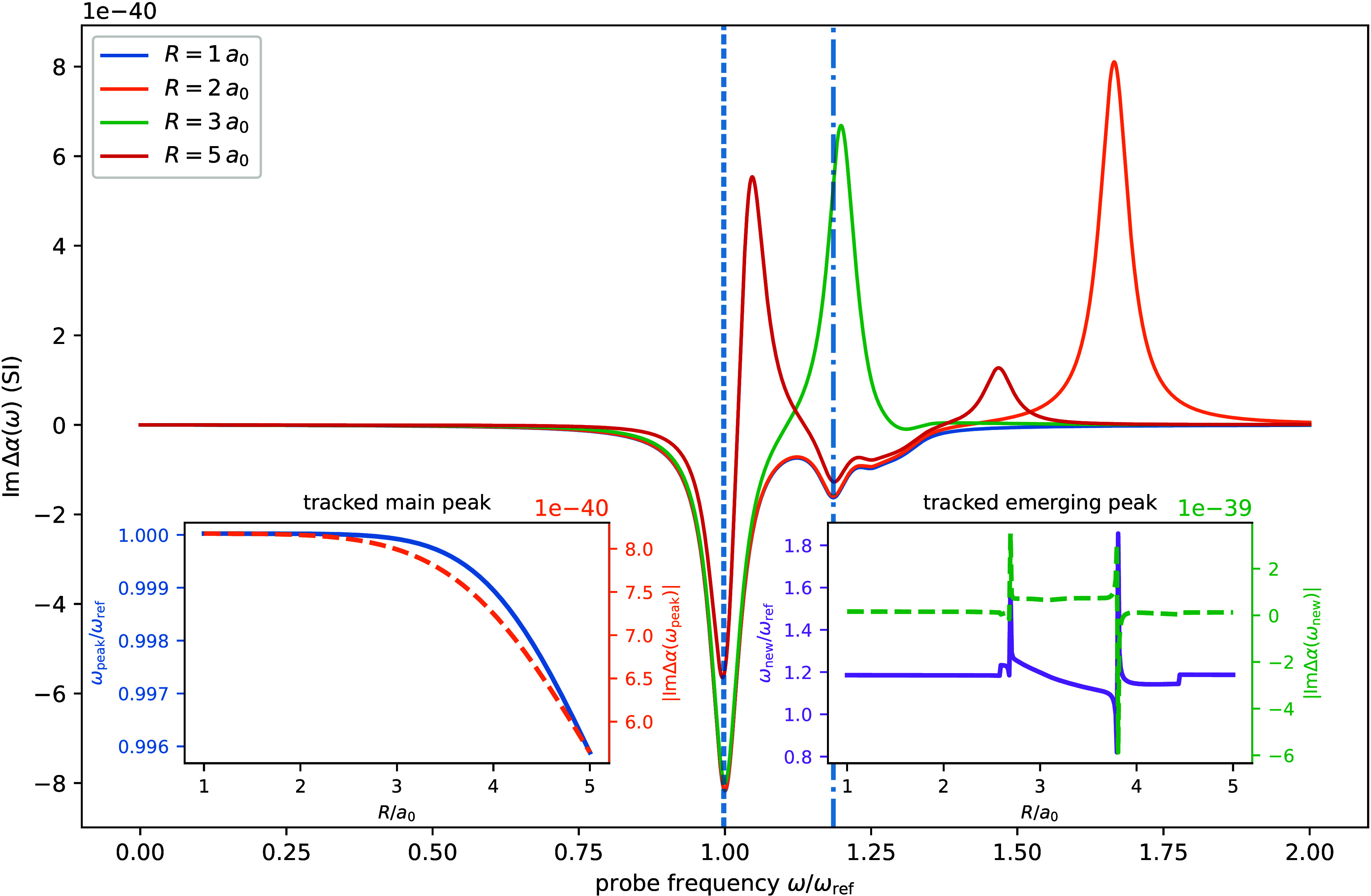
Imaginary part of the cavity-induced polarizability correction *Im* Δα­(ω) for the hydrogen ground state 1s for selected cavity radii *R*. Dashed vertical markers indicate the tracked main resonance near ω_ref_, while the dash-dotted marker highlights an emerging secondary resonance. Insets: (left) tracked main peak position and amplitude versus the cavity radius *R*; (right) tracked position and amplitude of the emerging resonance versus cavity radius *R*.

In addition to the main resonance, Figure 5 reveals an additional absorptive feature at a frequency ω_new_, marked in the main panel and analyzed in the lower-right inset. Unlike the primary peak, this resonance is not present in the large-cavity limit. Instead, it emerges only within a finite window of cavity radii, becoming clearly visible for intermediate values *R* ≈ (2.8–3.8)*a*_0_ and disappearing again as the cavity is reduced further.

This behavior is highlighted in the inset, where both the position and the amplitude of the emerging resonance are tracked as functions of *R*. The nonmonotonic dependence on the cavity radius demonstrates that this feature cannot be interpreted as a simple shifted atomic transition. Rather, it arises from the radius-dependent restructuring of the effective poles in the sum-over-states expression for the polarizability, induced by the cavity-modified self-energy corrections.

Physically, the appearance and disappearance of the additional resonance reflect the fact that different dipole-coupled transition channels are renormalized by different amounts as the cavity size is varied. As a consequence, specific transitions may temporarily enter the spectroscopically accessible frequency window and contribute significantly to the absorptive response before being shifted out again for smaller radii.

##### 4.3.3.3 Polarizability on the Imaginary Axis and Positivity

Finally, we consider the polarizability on the imaginary-frequency axis, α­(*i*ξ), which plays a central role in dispersion interactions and provides a useful consistency check for passivity and stability of the response. Figure 6 shows α­(*i*ξ) for the 1s state for the same set of cavity radii, normalized to its static free-space value α(0). Across the investigated parameter range, the response remains non-negative and varies smoothly with ξ, indicating that the cavity-induced renormalization in the present (denominator-only) approximation does not introduce unphysical sign changes on the imaginary axis. In the context of the PCM-inspired picture adopted here, this supports the interpretation that the cavity primarily renormalizes the effective transition frequencies and oscillator strengths in a manner consistent with a passive dielectric environment.

**Figure 6:**
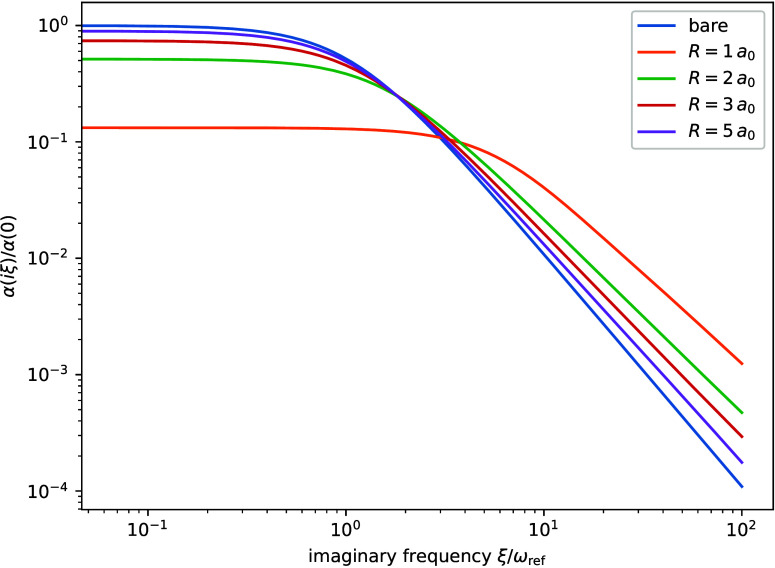
Hydrogen 1s polarizability on the imaginary axis, α­(*i*ξ), for selected cavity radii, normalized to the free-space static value α(0). No negativity is observed over the shown range of ξ/ω_ref_.

The existence of such transient, geometry-induced resonances highlights that cavity confinement can qualitatively alter the linear optical response of even the simplest atomic system beyond a rigid Lamb shift of isolated transitions.

## 5 Results and Discussion

The results presented above demonstrate that, even for the paradigmatic hydrogen atom, cavity confinement within a polarizable continuum leads to qualitative modifications of its electromagnetic response that go beyond a rigid Lamb shift of isolated energy levels. While the underlying mechanism is rooted in well-established macroscopic QED, the present calculations highlight how geometry-induced self-energy corrections propagate into experimentally relevant observables such as absorption spectra and dynamic polarizabilities.

### 5.1 Cavity-Induced Self-Energy: Magnitude and Scaling

The calculated level shifts shown in Figure 4 confirm the expected nonretarded scaling Δ*E* ∝ *R* ^–3^ for an atom located at the center of a spherical cavity. Within the physically motivated PCM range *R* ≃ (1–3)*a*_0_, the predicted shifts reach magnitudes comparable to optical transition frequencies. Although the absolute values should not be overinterpreted quantitatively, their scale alone demonstrates that cavity-induced self-energy effects cannot be regarded as small perturbations in this regime.

Importantly, different hydrogenic states experience markedly different shifts, reflecting the structures of their dipole-coupled manifolds. This state dependence implies that cavity confinement generically modifies transition frequencies nonuniformly, thereby reshaping the internal level structure rather than inducing a global energy offset. This observation forms the basis for all of the subsequent response effects discussed below.

### 5.2 Renormalization of the Absorptive Response

The consequences of the cavity-induced self-energy become particularly transparent when analyzing the frequency-dependent polarizability. As shown in Figure 5, the imaginary part of the correction Δα­(ω) exhibits two distinct effects.

First, the dominant absorptive resonance associated with the primary dipole-allowed transition is shifted in frequency in accordance with the cavity-modified transition energy. The tracked peak position varies smoothly with the cavity radius, providing a direct spectroscopic manifestation of the cavity-induced Lamb’s shift. Simultaneously, the peak amplitude is modified, indicating that the cavity not only shifts resonances but also redistributes the oscillator strength within the response function.

Second, an additional absorptive feature emerges that is absent in the large cavity limit. This resonance appears only within a finite interval of cavity radii, reaches a maximum strength for intermediate confinement, and disappears again as the cavity radius is reduced further. Such nonmonotonic behavior cannot be explained by a simple rigid shift of an isolated atomic line. Instead, it reflects the radius-dependent restructuring of the effective poles in the sum-over-states representation of the polarizability, caused by state-specific self-energy corrections.

From a physical perspective, this behavior arises because different dipole-coupled transitions are renormalized differently as the cavity geometry is varied. As a result, certain transitions may temporarily enter the spectroscopically accessible frequency window and contribute significantly to the absorptive response before being shifted out of it again. The cavity thus acts as a geometric control parameter that can selectively activate or suppress the specific response channels.

### 5.3 Imaginary-Frequency Response and Consistency

The behavior of the polarizability on the imaginary frequency axis provides an important consistency check. As shown in Figure 6, the cavity-renormalized α­(*i*ξ) remains non-negative and smoothly varies for all investigated radii. This indicates that, within the present denominator-renormalization approximation, the cavity-induced modifications preserve the passivity and stability of the linear response.

From the perspective of dispersion interactions, this result suggests that cavity-induced self-energy effects can be incorporated into effective polarizability models without introducing unphysical artifacts on the imaginary axis. This observation is particularly relevant for applications in which dispersion forces are evaluated by using Matsubara-frequency formulations.

### 5.4 Implications and Outlook

Although the present study focuses on hydrogen as the simplest possible test system, the underlying mechanism is entirely general. Any polarizable particle embedded in a structured electromagnetic environment experiences geometry-dependent self-energy corrections that modify both its energy spectrum and its response functions. The hydrogen atom, therefore, serves as a transparent benchmark that allows these effects to be isolated and analyzed without additional complications from electronic correlation or molecular structure.

From a broader perspective, the results highlight a route toward environment-engineered response functions in which geometric confinement and material properties jointly determine effective transition energies and oscillator strengths. This concept is directly relevant for dense molecular environments, solvated systems, and nanostructured cavities, where traditional descriptions based on fixed molecular polarizabilities may become insufficient.

In future work, a particularly promising direction is the systematic inclusion of the cavity-induced state mixing discussed in Section 3, which leads to explicit corrections to dipole matrix elements and thus modifies the numerator structure of the polarizability. Combined with fully dispersive material models and more complex geometries, this opens the door to a unified geometry-aware description of molecular response functions.

Such a framework would enable predictive modeling of environment-induced modifications of optical spectra, dispersion forces, and radiative processes and provide a natural starting point for extending macroscopic QED concepts to chemically and biologically relevant systems.

## 6 Conclusion

We have investigated how cavity-induced self-energy corrections modify the dynamic polarizability of an atom within the framework of macroscopic quantum electrodynamics. Using the hydrogen atom in a spherical vacuum cavity as a transparent benchmark system, we demonstrated that electromagnetic confinement does not merely shift atomic energy levels but can qualitatively reshape frequency-dependent response functions.

The cavity-induced Lamb shift leads to state-dependent renormalization of transition frequencies, which propagates into the sum-over-states expression for the polarizability. As a consequence, dominant absorptive resonances are shifted and redistributed and additional response features may appear within finite ranges of cavity radii. These geometry-induced resonances emerge from the collective restructuring of effective poles in the response function and are absent in the free space limit.

The behavior of the polarizability on the imaginary-frequency axis remains physically consistent, indicating that the present renormalization mechanism preserves the passivity and stability of the linear response. Although our analysis focuses on hydrogen and adopts a simplified cavity model, the underlying mechanism is entirely general and applies to any polarizable quantum system embedded in a structured electromagnetic environment.

Looking forward, the present work provides a conceptual foundation for extending polarizable continuum descriptions beyond fixed molecular response functions. The incorporation of environment-induced state mixing and fully dispersive material models opens the door to geometry-aware descriptions of molecular spectra, dispersion interactions, and radiative processes. Such effects are expected to become particularly relevant in dense molecular environments, nanocavities, and solvated systems, where electromagnetic confinement and material response act on comparable length scales.
